# Gaseous Elemental Mercury (GEM) Emissions from Snow Surfaces in Northern New York

**DOI:** 10.1371/journal.pone.0069342

**Published:** 2013-07-12

**Authors:** J. Alexander Maxwell, Thomas M. Holsen, Sumona Mondal

**Affiliations:** 1 Institute for a Sustainable Environment, Clarkson University, Potsdam, New York, United States of America; 2 Department of Civil and Environmental Engineering, Clarkson University, Potsdam, New York, United States of America; 3 Department of Mathematics, Clarkson University, Potsdam, New York, United States of America; University of Kansas, United States of America

## Abstract

Snow surface-to-air exchange of gaseous elemental mercury (GEM) was measured using a modified Teflon fluorinated ethylene propylene (FEP) dynamic flux chamber (DFC) in a remote, open site in Potsdam, New York. Sampling was conducted during the winter months of 2011. The inlet and outlet of the DFC were coupled with a Tekran Model 2537A mercury (Hg) vapor analyzer using a Tekran Model 1110 two port synchronized sampler. The surface GEM flux ranged from −4.47 ng m^−2^ hr^−1^ to 9.89 ng m^−2^ hr^−1^. For most sample periods, daytime GEM flux was strongly correlated with solar radiation. The average nighttime GEM flux was slightly negative and was not well correlated with any of the measured meteorological variables. Preliminary, empirical models were developed to estimate GEM emissions from snow surfaces in northern New York. These models suggest that most, if not all, of the Hg deposited with and to snow is reemitted to the atmosphere.

## Introduction

Hg is a potent neurotoxin and regulated by the U.S. EPA [1], European Union Restriction of Hazardous Substances Directive (RoHS) [2], and other government agencies worldwide as a hazardous pollutant. In the form of monomethylmercury (MeHg) it can adversely impact the development and health of both humans and wildlife [3]. Gaseous elemental mercury (GEM) is emitted into the atmosphere from both natural and anthropogenic sources, and has an atmospheric residence time of 0.5–2 years, allowing it to be transported over great distances [4]–[6]. Anthropogenic sources can also emit Hg in the form of gaseous oxidized Hg (GOM) and particulate bound Hg (PBM), which have shorter atmospheric lifetimes on the order of days to weeks [4]. GOM is fairly soluble in water, thus allowing it to be readily deposited to terrestrial surfaces through wet deposition, including snow [4]–[6]. The Hg deposited with snow is then either quickly revolatilized back into the atmosphere or incorporated into the snowpack. Newly deposited Hg has been shown to preferentially revolatilize, depending on the deposition surface, in a process known as prompt recycling [7].

While the role of snow surfaces in Hg cycling has been widely studied in arctic regions [5], [8]–[11], much less is known about its importance in more temperate climates [12]–[14]. Hg is deposited to snowpacks through both wet (snow) and dry deposition. Once deposited on the snowpack surface, it has been shown that >50% of the Hg deposited is reemitted within the first 24 hours [8], [12]. This process is believed to be governed by photoinduced reduction of GOM to GEM. Hg in the snowpack is mainly found in the form of GOM dissolved in snow grains, while <1% remains trapped in the interstitial air as GEM [8]. Hg concentrations are known to decrease with depth [12] with the higher concentrations up to 1.5 ng m^−3^ (GEM) remaining on the surface [8].

In the arctic, the snow surface-to-air flux of Hg is mainly the result of a diurnal pattern of GEM production in the interstitial air near the surface of the snowpack during the daytime (∼15–50 ng m^−2^ hr^−1^), with little contribution from deeper snow layers [15], [16]. However, internal production of GEM increases slightly with higher temperatures and snowmelt [8]. Since this process has not been well studied in temperate climates, measurements of snow surface-to-air fluxes were made over the 2011 winter season in Potsdam, NY.

## Materials and Methods

### Site Description, Methods, and Materials

Flux measurements were conducted at an open field site located at the Potsdam Municipal Airport (Damon Field) in Potsdam, NY (44°40.41N, −74°57.06′W) near the Clarkson University Observatory. This site remains largely undisturbed throughout the year and has served as a background site for the New York State particulate matter (PM) monitoring network. Sampling periods were determined based on access to the site and snow conditions. Special considerations were made to ensure that the chamber was never buried in snow and that all inlets and outlets remained above the snow during sampling. Measurements were conducted on a concrete slab, isolating the snowpack from the soil surface.

Concentrations of GEM were measured using a DFC with a method previously described in Choi & Holsen (2009). Briefly, the ambient sampling line (inlet) and chamber sampling line (outlet) of the DFC (described below) were coupled with a Tekran Model 2537A Hg vapor analyzer operated at room temperature in a field shed (Tekran Corporation, Inc., Toronto, Ontario, Canada) using a Tekran Model 1110 two-port synchronized sampler. The Tekran 1110 unit allowed for alternating five minute sampling pairs to be made between the inlet and outlet sample lines every 20 minutes (trap A inlet, trap B inlet; trap A outlet, trap B outlet). During inlet sampling, outlet air is bypassed at the same 1 L min^−1^ flow rate as the Tekran Model 2537A to maintain a constant turnover time (TOT) of 0.78 minutes and an optimized flushing flow rate (FFR) of 5 L min^−1^ [17] through the flux chamber. The inlet and outlet openings were placed next to each other at the same height, roughly 2 cm above the snow surface. Four, 1 cm diameter holes were evenly distributed around the perimeter of the chamber wall to insure the chamber was well-mixed. Although a standard method for the use of DFCs does not exist and this method has not been used in other snow studies, this sampling approach is similar to methods used in past studies over soil surfaces [17]–[19]. The 5 L min^−1^ FFR and 0.78 minute TOT are also similar to those used in a study by Eckley, et al. (2010).

**Figure 1:**
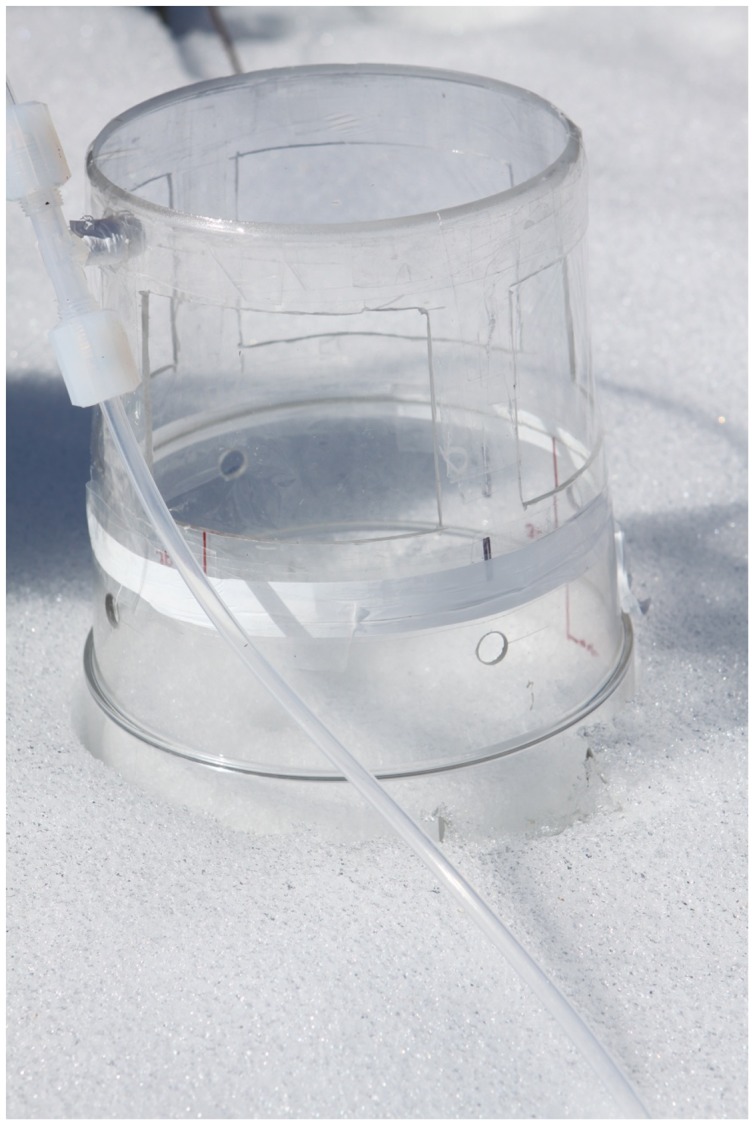
Modified Teflon Fluorinated Ethylene Propylene (FEP) Chamber with Polycarbonate (PC) Frame and 25 µm Teflon FEP Film Top and Side Windows.

Modified Teflon fluorinated ethylene propylene (FEP) chambers were used in the study. The modified Teflon chamber was constructed using a polycarbonate (PC) chamber frame and thin, 25 µm thick Teflon FEP film (CS Hyde Company, Lake Villa, IL) to cover the top and side windows (Figure 1). In previous studies [20], [21], Teflon film was shown to allow better UV permeability, up to 85±11% of light for wavelengths between 260 and 970 nm. Each DFC had a chamber volume of 3.9 L with a 18 cm diameter opening covering an area of approximately 254 cm^2^ of the snow surface.

Manual spike Hg recovery tests were conducted at the start of each sampling period by injecting 20 µL of Hg at roughly 13.23 pg µL^−1^ (20°C) or approximately 0.26 ng into an operating chamber using a calibrated (ANSI/NCSL Z540-1-1994) Hamilton Digital Syringe (Hamilton Company, Reno, NV) and a Tekran Model 2505 Hg vapor calibration unit (Tekran Corporation, Inc., Toronto, Ontario, Canada). The recorded Hg concentrations after each manual spike test were roughly 9 ng m^−3^, on the same order as the average daytime Hg concentrations around 2 ng m^−3^. The recovery was 97.5±3.8%. Flow rates were calibrated using a Bios Definer 220 volumetric flow meter (Bios International Corporation, Butler, NJ) at the beginning of each sampling period.

Prior to all field measurements, the Tekran Model 2537A was calibrated with an internal permeation source to ensure acceptable response factors (>6,000,000) and that the concentration difference between the inlet and outlet samples was less than 5%. In addition, all soda-lime traps and 0.2 µm polytetrafluoroethylene (PTFE) membrane filters were replaced at the start of each sampling period.

Meteorological data was collected using a weather station (Vantage Pro 2 Weather Station, Davis Instruments, Hayward, CA) located 1–2 m away from the chamber. The weather station measured ambient air temperature (°C), relative humidity (%), and solar radiation (W m^−2^) at a 10 minute time resolution.

### Sampling Analysis and Calculations

The GEM flux from the snow under the chamber was calculated using the following mass balance equation: 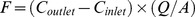
(1)


Where *F* is flux (ng GEM m^−2^ h^−1^); *C_outlet_* and *C_inlet_* are the concentrations of GEM (ng GEM m^−3^) at the outlet and inlet, respectively; *Q* is the FFR (m^3^ h^−1^) through the chamber; and *A* is the surface area (m^2^) of the snow exposed in the chamber. When fluxes were negative (-), Hg was being deposited on the snow surface, and when fluxes were positive (+) Hg was being emitted from the snow surface. All flux data was then smoothed using a Savitzky-Golay smoothing filter [22], (Eqn 2), to account for random error/noise while also preserving the quantitative information and trends.
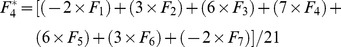
(2)


Where F_4_^*^ is the smoothed flux (ng GEM m^−2^ h^−1^), *F_1–7_* are the range of measured abscissa flux values (ng GEM m^−2^ h^−1^), and 21 is the normalizing factor.

**Figure 2:**
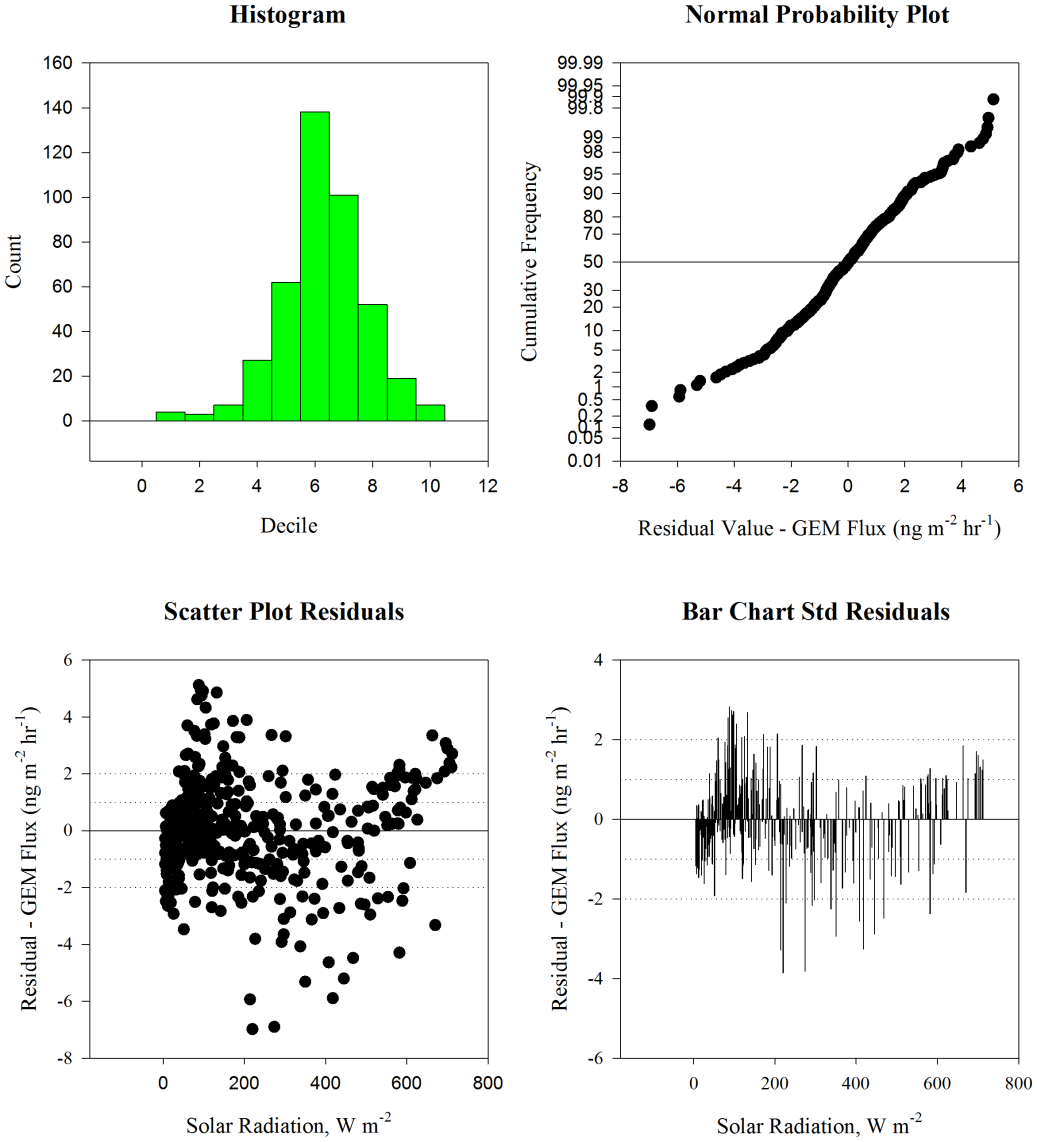
Histograms and Residual Plots of Daytime GEM Flux and Solar Radiation During Winter 2011.

Histograms of the GEM flux and the three meteorological predictor variables (temperature, solar radiation, and relative humidity) showed that none of the variables were normally distributed. Figure 2 provides histograms and residual plots of daytime GEM flux when compared to solar radiation. Similar plots were constructed for each individual variable, temperature, solar radiation, and relative humidity. Shapiro-Wilk normality tests [23] were then employed to confirm that the data deviated from normality. Non-parametric Pearson product-moment tests were used to determine the correlation coefficients (PPMC) between the variables [24].

## Results and Discussion

### Flux Measurements

During the 2011 winter sampling season, the flux was measured over five sampling periods, each lasting from one to six days. The measured flux ranged from a minimum −4.47 to a maximum 9.89 ng GEM m^−2^ h^−1^ (Table 1). The average daytime flux was 2.37±2.48 ng GEM m^−2^ h^−1^and the average nighttime flux was −0.35±0.41 ng GEM m^−2^ h^−1^. Measured nighttime Hg emission fluxes from other snowpack studies have been ≈0 ng GEM m^−2^ h^−1^ [8], while daytime fluxes have been shown to be much higher, ≈30–50 ng GEM m^−2^ h^−1^ [8]. Daytime fluxes were strongly correlated with solar radiation (PPMC value  = 0.684, p-value = 0.000<0.050) and to a lesser extent temperature and relative humidity (PPMC value  = 0.103, p-value = 0.035<0.050 and PPMC value  = −0.385, p-value = 0.000<0.050 respectively) (Table 2). This strong correlation with solar radiation suggests that the daytime Hg emissions from the snow surface are a result of the photoreduction of GOM associated with the snow to GEM. Similar results have been reported in Ferrari, et al. (2005), where it was also reported that GEM emissions from the snowpack were negligible in comparison to emissions caused by solar irradiation at the surface. Nighttime fluxes were only weakly correlated with both temperature (PPMC value  = −0.222, p-value = 0.000<0.050) and relative humidity (PPMC value  = −0.132, p-value = 0.002<0.000) (Table 2) and showed a statistically significant difference from zero.

**Table 1:** Measured Daytime And Nighttime GEM Flux Over 5 Winter 2011 Sampling Periods.

Date	Diurnal Period	GEM Flux (ng m^−2^ hr^−1^)					
		Mean	Std. Dev.	Range	Max	Min	Median
*21*–*24 Jan*	Daytime	1.13	1.37	5.60	4.44	−1.16	0.94
	Nighttime	−0.44	0.29	1.47	0.14	−1.33	−0.43
*26–31 Jan*	Daytime	2.65	1.92	8.20	6.57	−1.63	2.69
	Nighttime	−0.21	0.42	1.95	0.69	−1.26	−0.22
*15–18 Feb*	Daytime	1.88	2.96	12.56	8.09	−4.47	1.30
	Nighttime	−0.57	0.45	2.10	0.39	−1.71	−0.50
*23–24 Feb*	Daytime	3.03	2.60	9.38	8.13	−1.25	2.46
	Nighttime	−0.29	0.23	1.00	0.25	−0.75	−0.30
*08–09 Mar*	Daytime	3.50	3.08	9.97	9.89	−0.08	2.29
	Nighttime	−0.08	0.22	0.87	0.27	−0.60	−0.10
*Overall*	Daytime	2.37	2.48	14.36	9.89	−4.47	1.89
	Nighttime	−0.35	0.41	2.41	0.69	−1.71	−0.33

**Table 2:** Pearson Product-Moment Correlation Coefficients and P-Values For Correlations Between GEM Flux and Temperature, Relative Humidity, and Solar Radiation.

Date	Diurnal Period	Temperature (°C)		Relative Humidity (%)		Solar Radiation (W m^−2^)	
		Coefficient	P Value	Coefficient	P Value	Coefficient	P Value
*21–24 Jan*	Daytime	0.562	0.000	−0.494	0.000	0.546	0.000
	Nighttime	−0.004	0.959	−0.192	0.026	−	−
*26–31 Jan*	Daytime	0.189	0.022	−0.046	0.585	0.304	0.000
	Nighttime	−0.183	0.009	−0.319	0.000	−	−
*15–18 Feb*	Daytime	−0.518	0.000	0.673	0.000	0.820	0.000
	Nighttime	−0.553	0.000	−0.600	0.000	−	−
*23–24 Feb*	Daytime	0.300	0.027	−0.629	0.000	0.875	0.000
	Nighttime	0.000	0.997	−0.053	0.745	−	−
*08–09 Mar*	Daytime	0.446	0.001	−0.787	0.000	0.942	0.000
	Nighttime	−0.518	0.000	0.251	0.129	−	−
*Overall*	Daytime	0.103	0.035	−0.385	0.000	0.684	0.000
	Nighttime	−0.222	0.000	−0.132	0.002	−	−

**Figure 3:**
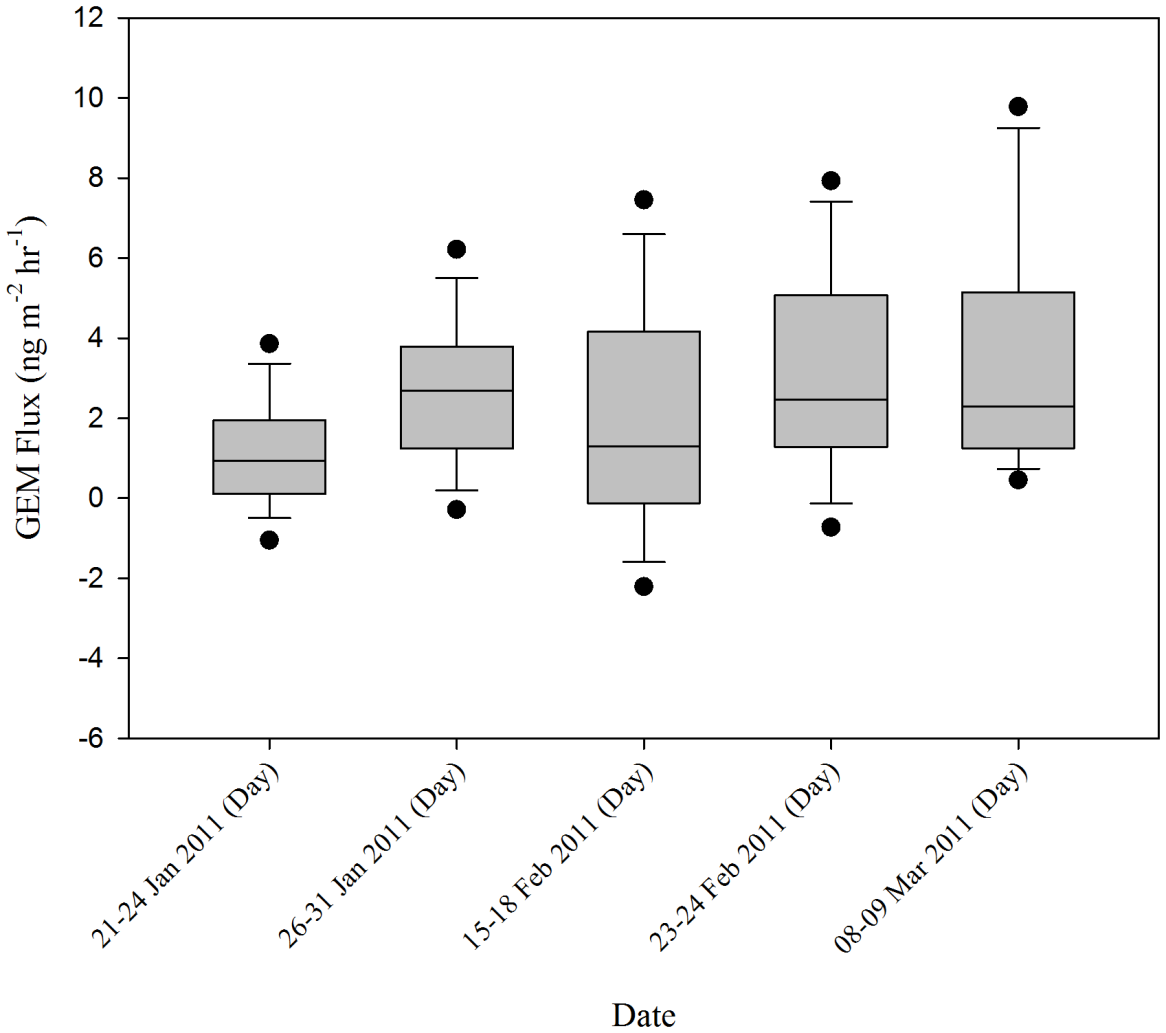
Average Daytime GEM Flux Measurements Made For Each Sampling Conducted During Winter 2011.

**Figure 4:**
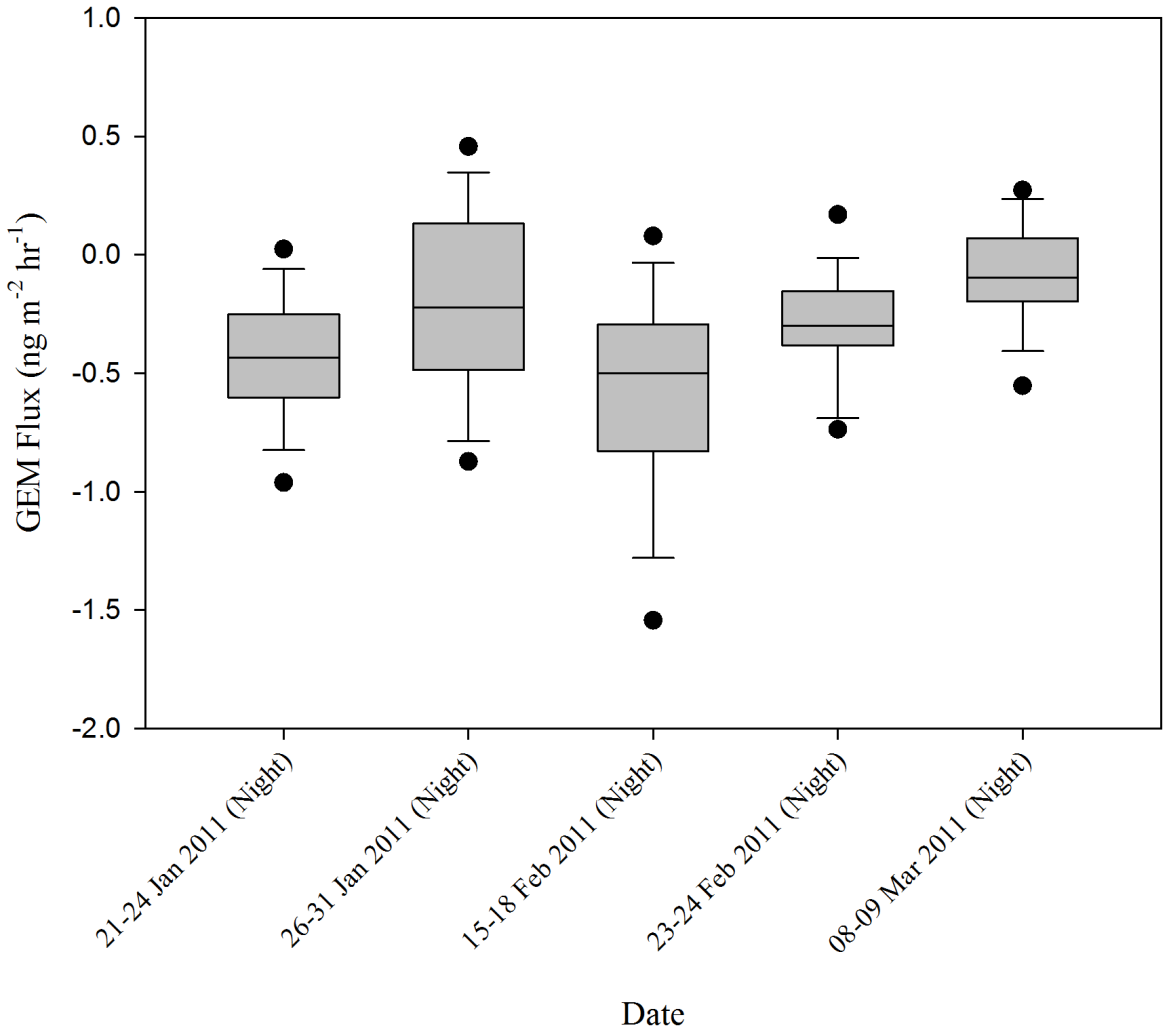
Average Nighttime GEM Flux Measurements Made For Each Sampling Conducted During Winter 2011.

**Figure 5:**
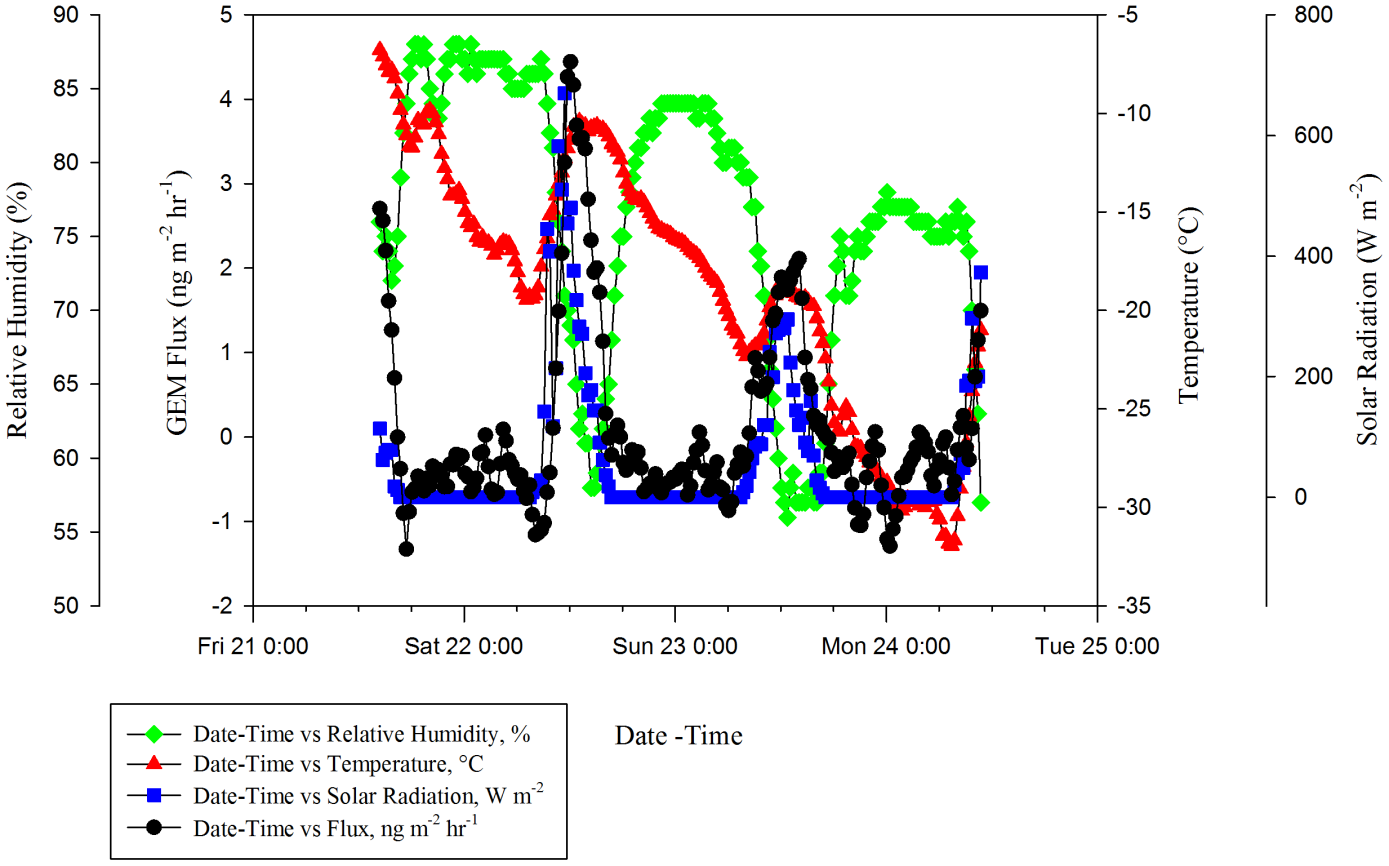
Diurnal Pattern Of GEM Flux For 21–24 January 2011 Sampling With Temperature, Relative Humidity, And Solar Radiation.

Overall, peak fluxes tended to increase later in the sampling season (Figure 3 and Figure 4). Emissions were highest during the last sampling period, 08–09 March, corresponding with the highest solar radiation peak (Max: 712 W m^−2^). Fluxes also tended to follow a diurnal pattern (Figure 5) with peaks occurring during the day following increased exposure to solar radiation, and deposition occurring at night, similar to patterns reported in other literature [15], [16].

### Impact of Solar Radiation

**Figure 6:**
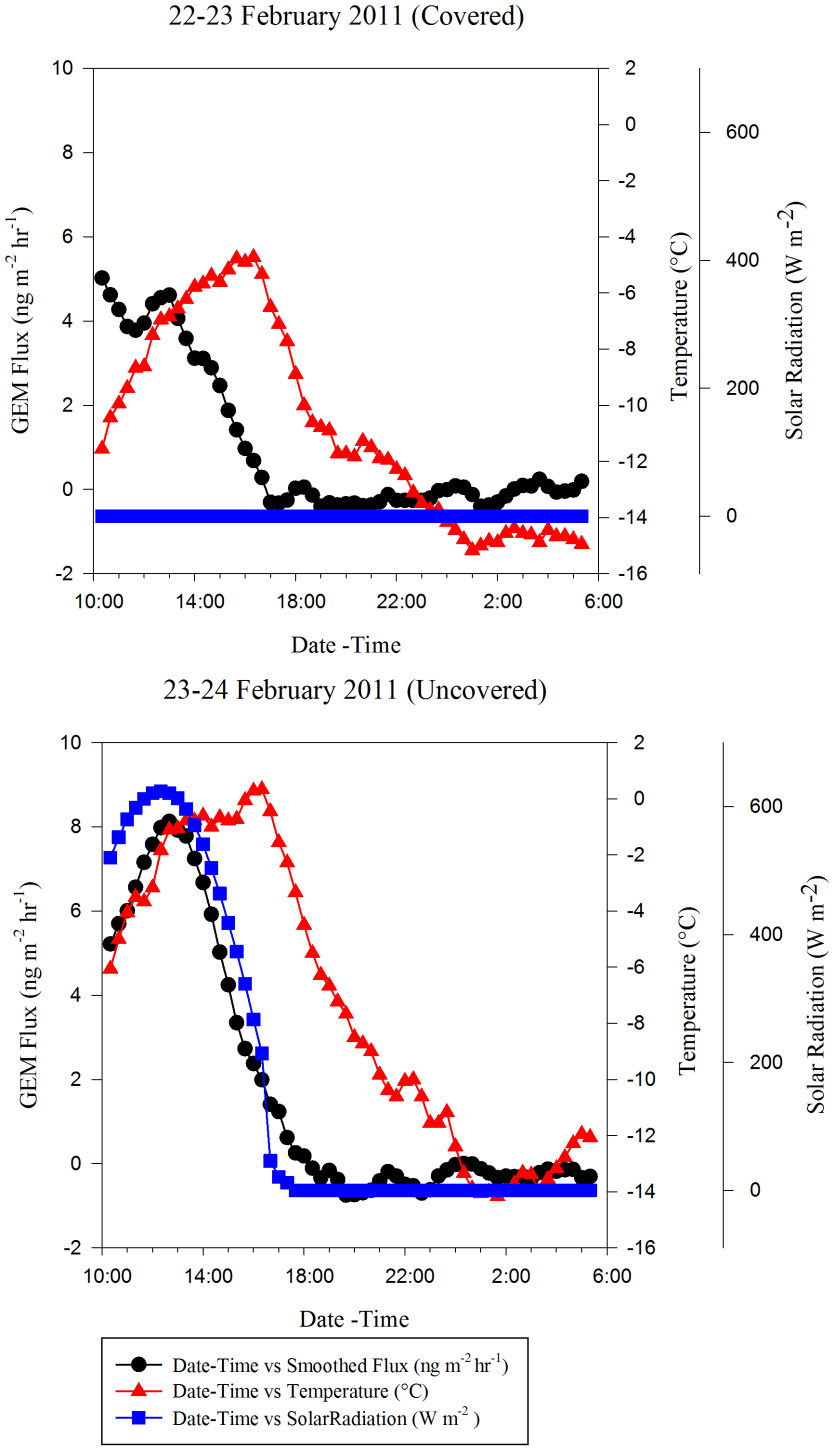
GEM Fluxes Measured Using Covered And Uncovered Chambers To Determine The Impact Of Solar Radiation On GEM Flux.

To test the impact of solar radiation on GEM fluxes, the chamber was covered with aluminum foil to simulate zero UV conditions. The uncovered measurements were made on 23–24 February 2011, while the covered measurements were made on 22–23 February 2011 (Figure 6). During the uncovered and covered tests, the average GEM fluxes were 1.76±3.06 and 0.99±1.81 ng GEM m^−2^ h^−1^ respectively. The covered DFC daytime measurements were negatively correlated with temperature (PPMC coefficient  = −0.624, p-value = 0.000<0.050) (Table 3). The slow decline in GEM flux after covering the chamber is likely a result of diffusion of GEM from the interstitial air in the snowpack into the DFC. The uncovered DFC daytime measurements were positively correlated with solar radiation, and to a lesser degree, temperature (PPMC coefficient  = 0.875, p-value = 0.000<0.050 and PPMC coefficient  = 0.300, p-value = 0.027<0.050) (Table 3), similar to what has been reported in other arctic studies [8]. Overall, solar radiation had the highest positive impact on GEM emissions, and though temperature and relative humidity were correlated to GEM flux, their correlation with solar radiation (PPMC coefficient  = 0.711 & −0.686 respectively, p-value = 0.000<0.050) indicate that their influence was likely a result of their codependence on solar radiation.

**Table 3:** PPMCs For Covered And Uncovered Chamber Tests For Impact Of Solar Radiation On GEM Flux.

Date	Diurnal Period	Temperature (°C)		Relative Humidity (%)		Solar Radiation (W m^−2^)	
		Coefficient	P Value	Coefficient	P Value	Coefficient	P Value
*22–23 Feb (Covered)*	Daytime	−0.624	0.002	0.489	0.021	−	−
	Nighttime	−0.374	0.025	0.141	0.412	−	−
*23–24 Feb (Uncovered)*	Daytime	0.300	0.027	−0.629	0.000	0.875	0.000
	Nighttime	0.000	0.997	−0.053	0.745	−	−

### Modeling

In the past, empirical models have been developed using meteorological data in order to estimate surface GEM flux from soils in temperate regions of eastern North America [17], [21], [25]. However, no model exists to estimate GEM flux from snow in the temperate climate of northern New York. Previous models for this region [17] excluded winter fluxes from snow surfaces. In order to better model GEM flux throughout the winter season, two multiple linear regression models were developed based on aggregated seasonal flux data:

Winter 2011 (Daytime): (R^2^ = 0.481)
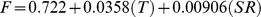



**Figure 7:**
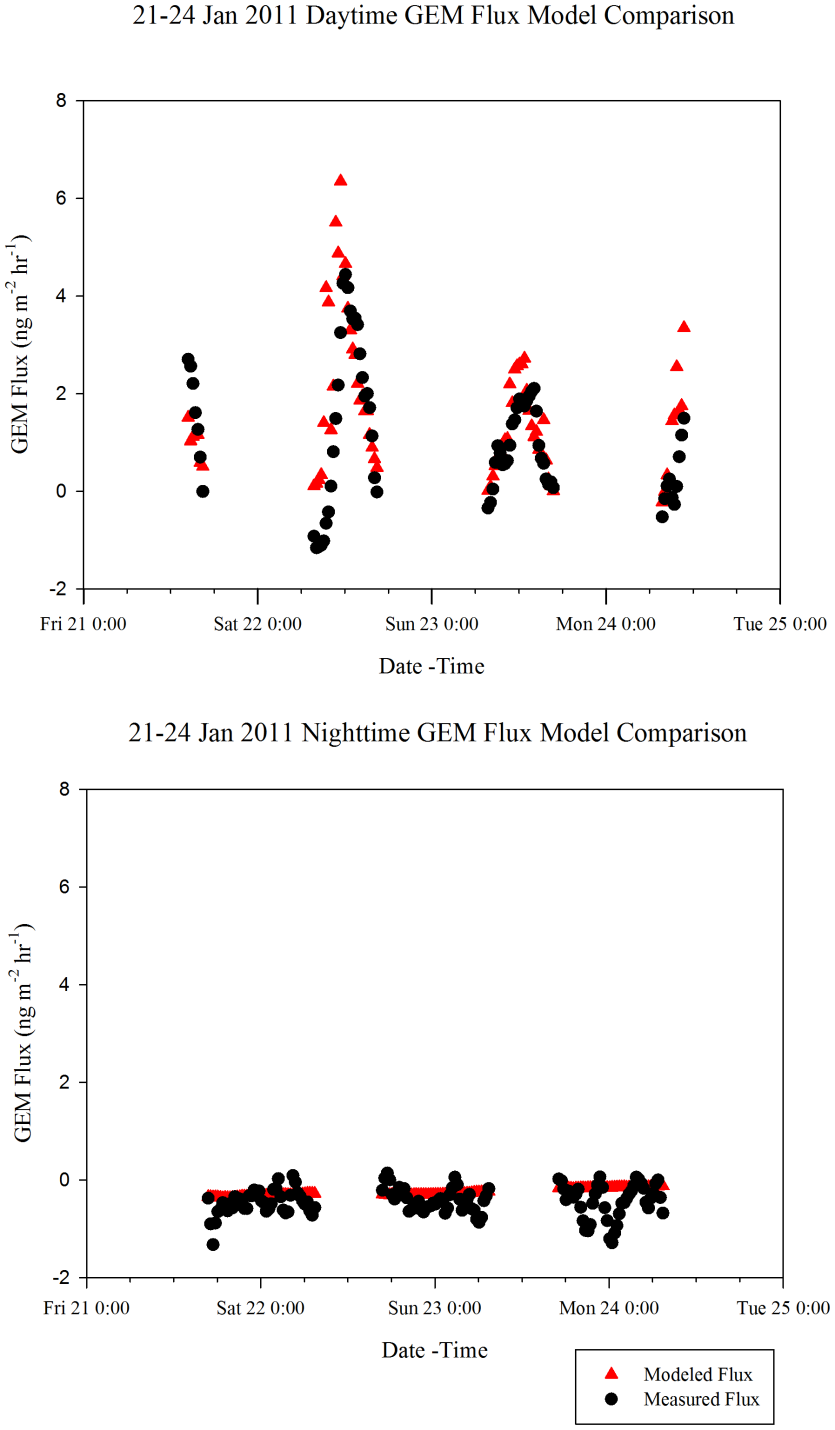
Daytime And Nighttime GEM Flux Model Comparison For 21–24 January Sampling Period.

Winter 2011 (Nighttime): (R^2^ = 0.0616)
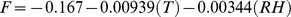



where *F* is GEM flux in ng m^−2^hr^−1^, *T* is ambient temperature in °C, *RH* is relative humidity in %, and *SR* is solar radiation in W m^−2^. Fluxes predicted by this model for the 22–24 January sampling period are shown in Figure 7.

Several nonlinear polynomial and power equation fits and variable transformations were conducted using SigmaPlot, ver. 12 in order to develop a more precise correlative model structure. However, the dynamic fits showed little improvement.

Using the multiple linear regression models in conjunction with 5-year winter (December-March, 2005–2010) EPA Clean Air Status and Trends Network (CASTNET) meteorological data from the National Atmospheric Deposition Program (NADP) site, NY20, it is estimated that the average snow surface emissions from the open Huntington Wildlife Forest (HWF) site range from −0.10±0.07 ng m^−2^ hr^−1^ (nighttime) to 1.53±1.69 ng m^−2^ hr^−1^ (daytime) or ∼17.22 ng m^−2^ year^−1^. During the same time period Mercury Deposition Network (MDN) data from the same site yield a similar value with an average deposition flux of 0.48±0.41 ng m^−2^ hr^−1^ or 11.52±9.84 ng m^−2^ year^−1^. The reason for the slightly higher modeled flux compared to the measured flux is likely due to the fact that some of the measurements used to make the empirical model were made after fresh snowfall when GEM fluxes would be at their maximum values.

Overall, these models suggest that most if not all the Hg deposited to snow surfaces is promptly recycled. Similar reemission phenomena have been reported by other research groups [8], [12] with mean emission fluxes of 2–5 ng m^−2^ hr^−1^, zero change in surface snow Hg concentration after deposition events, and up to 54% GEM reemission during the first 24 hours after a snowfall.

### Deposition Events

**Figure 8:**
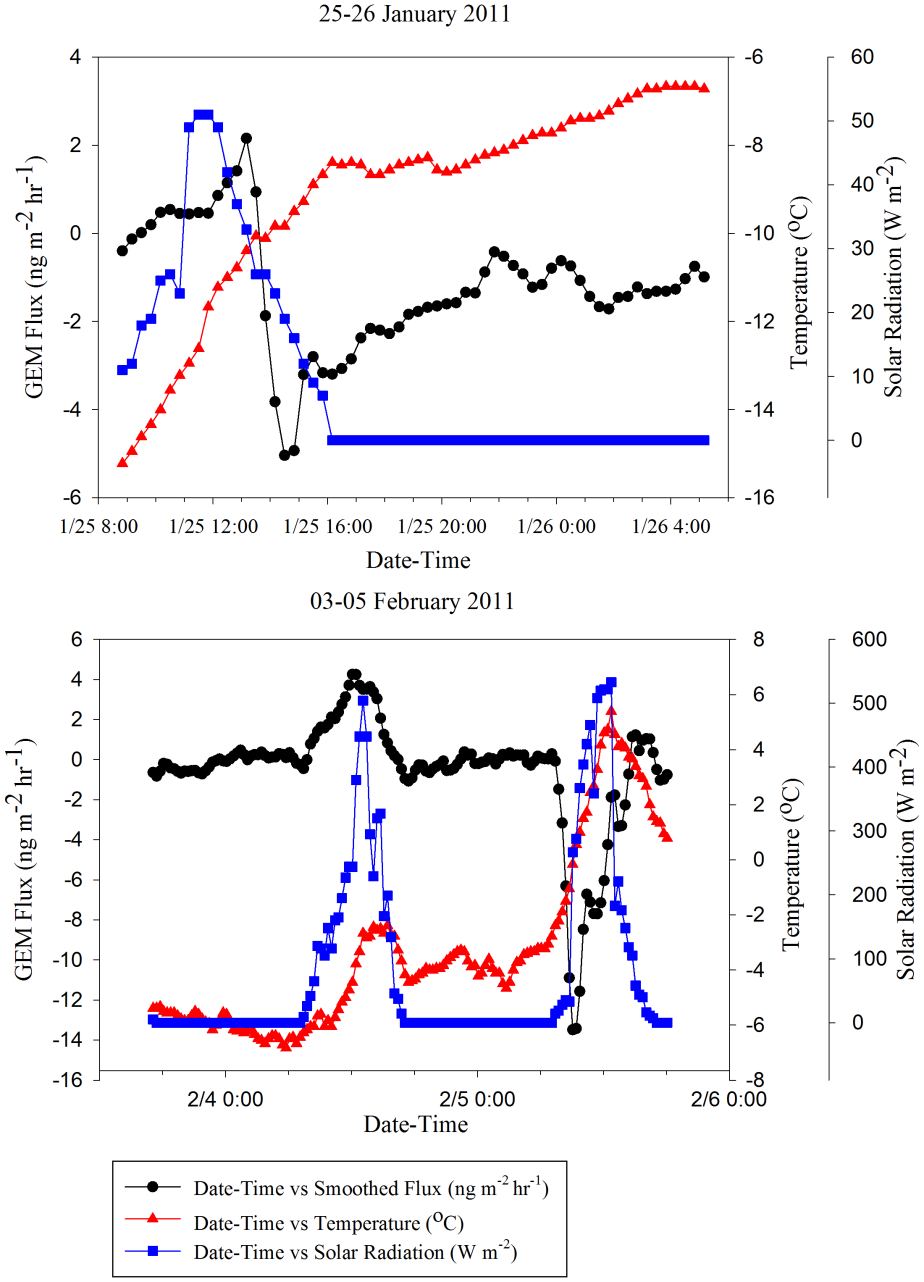
GEM Deposition Events For 25–26 January And 03–05 February 2011 Sampling Period.

Two unique deposition events with fluxes as high as −14 ng m^−2^ hr^−1^ occurred during separate sampling periods, one on 25 January and one on 05 February. Both of these event followed snowfalls ≥3 cm (Table 4) and melting also occurred during the 03–05 February sampling (Figure 8). During both of these events, fluxes were negatively correlated with temperature (PPMC coefficient  = −0.421 and −0.439 respectively, p-value = 0.000<0.050) and displayed patterns opposite to the diurnal patterns typically seen. This sudden deposition event is similar to atmospheric Hg depletion events (AMDEs) witnessed in arctic regions during polar sunrise [9]–[11].

**Table 4:** Field Observations Made During Various Measurement Periods Throughout the 2011 Winter Sampling Season.

Date	Observation
*21–24 Jan.*	No recent snow
*26–27 Jan.*	Fresh snow (dusting, <2.5 cm) prior to sampling
*27–30 Jan.*	Fresh snow (dusting, <2.5 cm) and intermittent light snowing throughout sampling (no enough to cover inlets of chamber)
*30–31 Jan.*	No recent snow
*15–18 Feb.*	No recent snow
*23–24 Feb.*	No recent snow
*08–09 Mar.*	Fresh snow (≈7.5 cm); during end of season melt

During an AMDE, rapid oxidation of GEM forms GOM that is subsequently deposited to the snow surface. Arctic AMDEs are springtime phenomenon that occur as a result of reactions with ozone and other halogen compounds, especially bromine oxides [26]. Though the cause of the two deposition events seen in Potsdam is unclear, they could coincide with sudden increases in atmospheric oxidant concentrations including free halogens.

## Conclusions

Snow surface-to-air exchange of gaseous elemental Hg (GEM) was measured using a modified Teflon fluorinated ethylene propylene (FEP) dynamic flux chamber (DFC) in a remote, open site in Potsdam, New York during the winter months of 2011. The surface GEM flux ranged from −4.47 ng m^−2^ hr^−1^ to 9.89 ng m^−2^ hr^−1^. For most sample periods, the daytime GEM flux was strongly correlated with solar radiation. The average nighttime GEM flux was slightly negative and was weakly correlated with all of the measured meteorological variables. Overall, preliminary models indicate that most if not all the Hg being deposited to snow surfaces is being reemitted back into the atmosphere. Two unique deposition events with fluxes as high as −14 ng m^−2^ hr^−1^ occurred during separate sampling periods following snowfalls ≥3 cm. During both of these events, fluxes were negatively correlated (PPMC coefficient  = −0.421 and −0.439 respectively, p-value = 0.000<0.050) with temperature and displayed patterns opposite to the diurnal patterns typically seen.
